# Functional Recovery, Symptoms, and Quality of Life 1 to 5 Years After Traumatic Brain Injury

**DOI:** 10.1001/jamanetworkopen.2023.3660

**Published:** 2023-03-20

**Authors:** Lindsay D. Nelson, Nancy R. Temkin, Jason Barber, Benjamin L. Brett, David O. Okonkwo, Michael A. McCrea, Joseph T. Giacino, Yelena G. Bodien, Claudia Robertson, John D. Corrigan, Ramon Diaz-Arrastia, Amy J. Markowitz, Geoffrey T. Manley, and the TRACK-TBI Investigators

**Affiliations:** 1 Medical College of Wisconsin, Milwaukee; 2 University of Washington, Seattle; 3 University of Pittsburgh Medical Center, Pittsburgh, Pennsylvania; 4 Massachusetts General Hospital and Harvard Medical School, Boston; 5 Spaulding Rehabilitation Hospital, Charlestown, Massachusetts; 6 Baylor College of Medicine, Houston, Texas; 7 The Ohio State University Wexner Medical Center, Columbus; 8 University of Pennsylvania, Philadelphia; 9 University of California, San Francisco

## Abstract

**Importance:**

Many level I trauma center patients experience clinical sequelae at 1 year following traumatic brain injury (TBI). Longer-term outcome data are needed to develop better monitoring and rehabilitation services.

**Objective:**

To examine functional recovery, TBI-related symptoms, and quality of life from 1 to 5 years postinjury.

**Design, Setting, and Participants:**

This cohort study enrolled trauma patients across 18 US level I trauma centers between 2014 and 2018. Eligible participants were enrolled within 24 hours of injury and followed up to 5 years postinjury. Data were analyzed January 2023.

**Exposures:**

Mild TBI (mTBI), moderate-severe TBI (msTBI), or orthopedic traumatic controls (OTC).

**Main Outcomes and Measures:**

Functional independence (Glasgow Outcome Scale-Extended [GOSE] score 5 or higher), complete functional recovery (GOSE score, 8), better (ie, lower) TBI-related symptom burden (Rivermead Post Concussion Symptoms Questionnaire score of 15 or lower), and better (ie, higher) health-related quality of life (Quality of Life After Brain Injury Scale-Overall Scale score 52 or higher); mortality was analyzed as a secondary outcome.

**Results:**

A total 1196 patients were included in analysis (mean [SD] age, 40.8 [16.9] years; 781 [65%] male; 158 [13%] Black, 965 [81%] White). mTBI and OTC groups demonstrated stable, high rates of functional independence (98% to 100% across time). While odds of independence were lower among msTBI survivors, the majority were independent at 1 year (72%), and this proportion increased over time (80% at 5 years; group × year, *P* = .005; independence per year: odds ratio [OR] for msTBI, 1.28; 95% CI, 1.03-1.58; OR for mTBI, 0.81; 95% CI, 0.64-1.03). For other outcomes, group differences at 1 year remained stable over time (group × year, *P* ≥ .44). Odds of complete functional recovery remained lower for persons with mTBI vs OTC (OR, 0.39; 95% CI, 0.28-0.56) and lower for msTBI vs mTBI (OR, 0.34; 95% CI, 0.24-0.48). Odds of better TBI-related symptom burden and quality of life were similar for both TBI subgroups and lower than OTCs. Mortality between 1 and 5 years was higher for msTBI (5.5%) than mTBI (1.5%) and OTC (0.7%; *P* = .02).

**Conclusions and Relevance:**

In this cohort study, patients with previous msTBI displayed increased independence over 5 years; msTBI was also associated with increased mortality. These findings, in combination with the persistently elevated rates of unfavorable outcomes in mTBI vs controls imply that more monitoring and rehabilitation are needed for TBI.

## Key Points

**Question:**

What is the course of functional, symptom, and quality of life outcomes 1 to 5 years after mild traumatic brain injury (mTBI) and moderate-severe traumatic brain injury (msTBI)?

**Findings:**

In this cohort study of 1196 level I trauma center patients (859 mTBI, 188 msTBI, 152 orthopedic trauma controls) followed 5 years postinjury, msTBI was associated with increased mortality, yet msTBI survivors displayed improved independence from 1 to 5 years. mTBI survivors had poorer outcomes than controls.

**Meaning:**

These results suggest that better understanding of TBI recovery, as well as increased clinical and community support for mTBI and msTBI, are warranted to address long-term risks associated with TBI.

## Teaser

This cohort study of level I trauma center patients compares outcomes for function and quality of life of patients with mild and moderate-severe traumatic brain injury over the course of 5 years following injury.

## Introduction

Clinical recovery from traumatic brain injury (TBI) typically proceeds most rapidly in the first 3 to 6 months postinjury.^1,2,3,4,5^ Most studies have terminated follow-up by 6 months, resulting in limited data on TBI’s long-term natural history.^6^ Smaller, retrospective studies and investigations of restricted subgroups (eg, patients admitted to inpatient rehabilitation) indicate that TBI recovery is dynamic, continuing for years. These findings counter the clinical narrative that recovery is unidirectional (ie, improving) and time-limited.^7,8,9,10,11^ Although many persons appear to have an ongoing need for support to overcome and compensate for TBI-related deficits, they rarely receive formal clinical services beyond the first few months postinjury.^12,13,14^ Large-scale prospective studies characterizing the long-term outcomes of TBI may inform initiatives to develop systems of postacute care for TBI that better meet the long-term needs of this population,^14^ while promoting clinical research aimed at developing disease-modifying therapies.

In recent years, data from large-scale prospective studies^5,8,15,16^ have enriched understanding of the natural history of TBI. The multicenter Transforming Research and Clinical Knowledge in TBI (TRACK-TBI) study revealed that, contrary to popular belief that mild TBI (mTBI) (ie, Glasgow Coma Scale [GCS] score, 13-15) is a time-limited condition, the majority of level I trauma center patients with mTBI continue to report symptoms and injury-related problems with daily function at 1 year postinjury.^15,17^ Persons with moderate-severe TBI (msTBI) (GCS score, 3-12) in the TRACK-TBI sample have displayed varied outcomes, often achieving functional independence and returning to work but rarely making a complete recovery.^5^ Unlike functional outcomes, which show a clear dose-response association with TBI severity, TBI-related symptoms show elevated rates of persistence in all level I trauma center patients with TBI, with weak and sometimes conflicting associations with TBI severity.^17,18^ Similarly, survivors of TBI often report satisfactory quality of life irrespective of their injury severity and functional limitations.^19,20,21^ These findings highlight the value of capturing differing outcome measures over time.

Our objective was to examine the degree to which TBI across severities is associated with functional recovery, TBI-related symptoms, and quality of life from 1 to 5 years postinjury. We hypothesized that outcomes would continue to be poorest through the follow-up period post-msTBI, followed by mTBI, followed by orthopedic trauma control (OTC) groups.

## Methods

### Participants and Study Design

This manuscript follows Strengthening the Reporting of Observational Studies in Epidemiology (STROBE) guidelines for reporting observational studies. TRACK-TBI is a prospective, multicenter study recruiting participants from 18 level I trauma centers. The study was approved by the institutional review board of each enrolling institution. Participants or their legally authorized representatives completed written informed consent. The initial study enrolled participants from February 26, 2014, to July 27, 2018, within 24 of hours of injury with follow-up until 12 months postinjury. The clinical outcomes of the sample at these time points have been reported elsewhere.^5,15,22^ In 2019, the study began TRACK-LONG, which aimed to conduct up to 3 annual phone calls for living participants at least 2 years postinjury. The eligible sample for the present study of longer-term clinical outcomes were TBI or OTC participants who were at least 17 years old, had a known admission GCS score, and were not known to have died by 1 year postinjury (eTable 1 in Supplement 1). Of these, primary analyses focused on the participants who were not known to have died through 5 years postinjury and who completed at least 1 primary outcome from 2 to 5 years postinjury. Secondary analyses of mortality included another 20 participants who died between 1 and 5 years postinjury.

### Inclusion Criteria

Inclusion criteria for TBI participants in the parent study were presentation within 24 hours of injury to a participating level I trauma center with clinical suspicion of TBI (ie, head computed tomography [CT] scan ordered) and either showing objective evidence of brain injury (eg, positive results from head CT) or displaying or reporting altered consciousness (American Congress of Rehabilitation Medicine TBI definition).^23^ OTCs presented within 24 hours of injury with orthopedic injuries and were excluded if they had physical signs of head trauma or met any criteria for TBI. Exclusions for all participants were being in custody of law enforcement, pregnancy, having nonsurvivable physical trauma, history of debilitating mental health disorders or neurological disease, and being non-English speaking (except for sites that enrolled Spanish-speaking individuals).

### Outcome Measures

#### Glasgow Outcome Scale-Extended

The Glasgow Outcome Scale-Extended (GOSE) estimates how injuries have affected daily function (eg, home independence and return to work, social, and other areas of preinjury daily functioning). Respondents (participants or informants) report on new or worsened difficulties since injury^24,25^; responses considered were given a score on an 8-point scale (with 1 indicating death; 2, vegetative state; 3, lower severe disability; 4, upper severe disability; 5, lower moderate disability; 6, upper moderate disability; 7, lower good recovery; and 8, upper good recovery). Deaths were identified through research assistants’ contact with secondary contacts when pursuing follow-up visits. For the present study, GOSE scores reflected disability due to TBI and accompanying peripheral injuries, when present. Based on nonlinearity of the scale and differing traditional cut points for mTBI and msTBI, we dichotomized the GOSE to reflect both functional independence (GOSE score 5 or above) and complete functional recovery (GOSE score of 8).

#### Rivermead Post Concussion Symptoms Questionnaire

The Rivermead Post Concussion Symptoms Questionnaire (RPQ) assesses the presence and severity of 16 symptoms that are new or worsened since the injury.^26^ These diverse physical, cognitive, and emotional symptoms occur in all populations,^27^ but are especially common after TBI.^28^ Injury-related symptoms are rated by participants as 2 (mild problem relative to preinjury), 3 (moderate), or 4 (severe); summed ratings yield a symptom severity score ranging from 0 to 64. Following a previously established threshold to project persistent postconcussion symptoms, we defined better (ie, lower) TBI symptom burden as RPQ of 15 or lower.^29,30^

#### Quality of Life After Brain Injury-Overall Scale

The 6-item Quality of Life After Brain Injury-Overall Scale (QOLIBRI-OS) assesses health-related quality of life (QoL) in domains of cognitive, emotional, and physical function^31^ on a 5-point scale (from not at all satisfied to very satisfied). Summed ratings (self-reported by participants) are transformed to produce a total score (100-point scale, with higher equaling better QoL). Scores were dichotomized as 52 or higher (better QoL) vs below 52 (worse QoL), a recommended cut score to indicate probable emotional distress and impaired health-related quality of life.^32,33^

### Statistical Analysis

Characteristics of subsamples with vs without outcome data were compared descriptively (percentages, means) and with Mann-Whitney *U* tests and Fisher exact tests. To facilitate drawing inferences for the full enrolled sample, all primary analyses incorporated inverse probability weighting. This approach more heavily weights data from groups who disproportionately did not have follow-up data to estimate parameters for the fully enrolled sample (eMethods in Supplement 1). Analyses were also performed without weighting, with a trivial effect on results.

Preliminary analyses also compared the mTBI, msTBI, and OTC groups on demographic and injury variables using Kruskal-Wallis H tests and Fisher exact tests to inform selection of covariates in multivariable regression models. For the primary analyses, we first computed the percentage of persons within each group who met each outcome at 1, 2, 3, 4, and 5 years. Second, we used mixed effects logistic regression models to evaluate the degree to which group, year since injury (treated linearly), and group × year estimated odds of each outcome, controlling for relevant sociodemographic and injury covariates (age, sex, race, ethnicity, insurance type, education, prior TBI, cause of injury). Categories for race included Alaskan Native or Inuit, Asian, Black, Native Hawaiian or Pacific Islander, Indian, White, and unknown; the source from which examiners collected racial and ethnic data was not documented. Groups with insufficient numbers of individuals who were functionally dependent (OTC group in primary analyses, and additionally the mTBI group without acute intracranial findings in supplemental analyses) were excluded from logistic regression analysis of functional independence (GOSE score 5 or above). Contrasts of within-group changes in outcome measures over time were calculated to interpret group × year interactions. Nonsignificant interaction terms were dropped to facilitate interpretability of main effects. In secondary analyses, we computed percentages for each GOSE domain to further describe the groups’ impairment or function. Supplemental analyses examined (1) the outcomes of the mTBI group stratifying the group by the presence or absence of acute intracranial findings on clinical head CT scans performed upon hospital admission and (2) outcomes for a subset of individuals who were followed from year 1 through year 4 or 5.

Finally, we computed cumulative mortality (ie, not known to have died at 1 year) for participants who died between 1 and 5 years postinjury, and compared groups on cumulative mortality using the log rank test. Tests were 2-tailed with α = .05; no adjustments were made for multiple comparisons. Inverse probability weights were derived using the TWANG Shiny App software package for Windows developed by RAND Corporation (downloaded in December 2019).^34^ Mixed-effects regression modeling was conducted using SAS statistical software version 9.4 (SAS Institute), and other statistical analyses were conducted in SPSS Statistics for Windows version 26 (IBM Corp).

## Results

### Sample Characteristics

The full enrolled sample included 2661 participants (mean [SD] age, 41.0 [17.0] years; 1814 male [68%]; 436 Black [16%], 2034 White [76%], 191 other [7%]); 1196 participants were in the sample with outcome data vs 1465 participants without outcome data (eTable 2 in Supplement 1). A total 856 individuals were in the mTBI group, 188 in the msTBI group, and 152 in the OTC group, with statistically significant differences in demographics (age, sex), socioeconomic factors (eg, insurance type, educational history), prior TBI history, and cause of injury (Table 1). The percentage of participants with acute intracranial findings on admission head CT scans was 39% (322 of 832) in the mTBI group and 91% (162 of 178) in the msTBI group.

**Table 1.** Sample Characteristics and Group Comparisons

Demographics	Patients, No. (%)			*P* value^a^
	msTBI (n = 188)	mTBI (n = 856)	OTC (n = 152)	
Age, mean (SD), y	35.5 (14.4)	41.9 (17.3)	41.4 (15.7)	<.001
Sex				
Female	45 (24)	309 (36)	61 (40)	.002
Male	143 (76)	547 (64)	91 (60)	
Race^b^				
Black	21 (11)	113 (13)	24 (16)	.59
White	152 (81)	693 (81)	120 (79)	
Other/unknown^c^	15 (8)	50 (6)	8 (5)	
Hispanic ethnicity	36 (19)	155 (18)	34 (23)	.40
Insurance				
Medicaid/uninsured	73 (40)	223 (26)	35 (23)	.001
Other insured^d^	110 (60)	619 (74)	115 (77)	
Education, mean (SD), y	13.0 (2.7)	14.0 (2.8)	14.4 (2.8)	<.001
Previous TBI				
No	152 (87)	636 (79)	117 (83)	.03
Yes, with hospitalization	16 (9)	102 (13)	20 (14)	
Yes, without hospitalization	6 (3)	63 (8)	4 (3)	
Neurodevelopmental disorder	13 (7)	72 (8)	11 (7)	.78
Mental health history	41 (22)	203 (24)	33 (22)	.80
Injury characteristics				
Injury cause				
MVC (occupant)	55 (29)	251 (29)	24 (16)	<.001
MCC	23 (12)	56 (7)	16 (11)	
MVC (cyclist or pedestrian)	26 (14)	151 (18)	10 (7)	
Fall	38 (20)	243 (28)	52 (34)	
Assault	12 (6)	46 (5)	2 (1)	
Other/unknown^e^	34 (18)	109 (13)	48 (32)	
CT results positive (vs negative)	162 (91)	322 (39)	0	<.001
Loss of consciousness^f^	178 (98)	706 (86)	0	<.001
Posttraumatic amnesia^f^	124 (94)	635 (80)	0	<.001
AIS head/neck ≥3	169 (90)	257 (30)	2 (1)	<.001
Maximum non-head/neck AIS ≥3	56 (30)	135 (16)	29 (19)	<.001
Highest level of care				
Emergency department	0	216 (25)	60 (39)	<.001
Inpatient unit	4 (2)	384 (45)	85 (56)	
Intensive care unit	184 (98)	256 (30)	7 (5)	
Injury-related litigation	32 (22)	159 (22)	18 (15)	.22

Abbreviations: AIS, Abbreviated Injury Severity score; CT, computed tomography; MCC, motorcycle crash; msTBI, moderate-severe traumatic brain injury; mTBI, mild traumatic brain injury; MVC, motor vehicle crash; OTC, orthopedic trauma control; TBI, traumatic brain injury.

^a^ Unweighted *P* values reported from Mann-Whitney and Fisher exact tests.

^b^ The source of race and ethnicity (eg, medical records vs participant report) was not collected.

^c^ Other or unknown race categories included Alaska Native or Inuit, Asian, American Indian, mixed race, Native Hawaiian or Pacific Islander, and unknown.

^d^ Other insurance categories included: insurance purchased directly from an insurance company or on the health insurance exchange (this person or family member); insurance through a current or former employer (of this person or another family member); Medicare, for people aged 65 years and older or people with certain disabilities; TRICARE (the US military’s health care program), Department of Veterans Affairs, or other military health care; and other.

^e^ Injury cause categories included act of mass violence, other, other nonintentional injury, other road traffic incident, suicide attempt, and unknown.

^f^ Witnessed and suspected categories collapsed.

### Prevalence and Group Differences in Clinical Outcomes from 1 to 5 Years Postinjury

Figure 1 illustrates the unweighted percentage (95% CI) of each primary outcome by group (eTable 3 for accompanying numbers, eTable 4 for primary analyses using propensity weighting, and eTable 5 for sensitivity analyses verifying results in a subset of individuals who were followed from year 1 to years 4 and 5 in Supplement 1). Table 2 provides results of propensity-weighted mixed effects logistic regression models evaluating the effects of group, year, and group × year on the odds of each outcome, controlling for several sociodemographic and injury variables. Covariates were selected because they demonstrated different distributions across groups (Table 1) or were of substantive interest as potential social determinants of TBI outcomes. Unadjusted, unweighted regression models revealed quite similar group and year effects as the more complex multivariable models (eTable 6 in Supplement 1).

**Figure.**
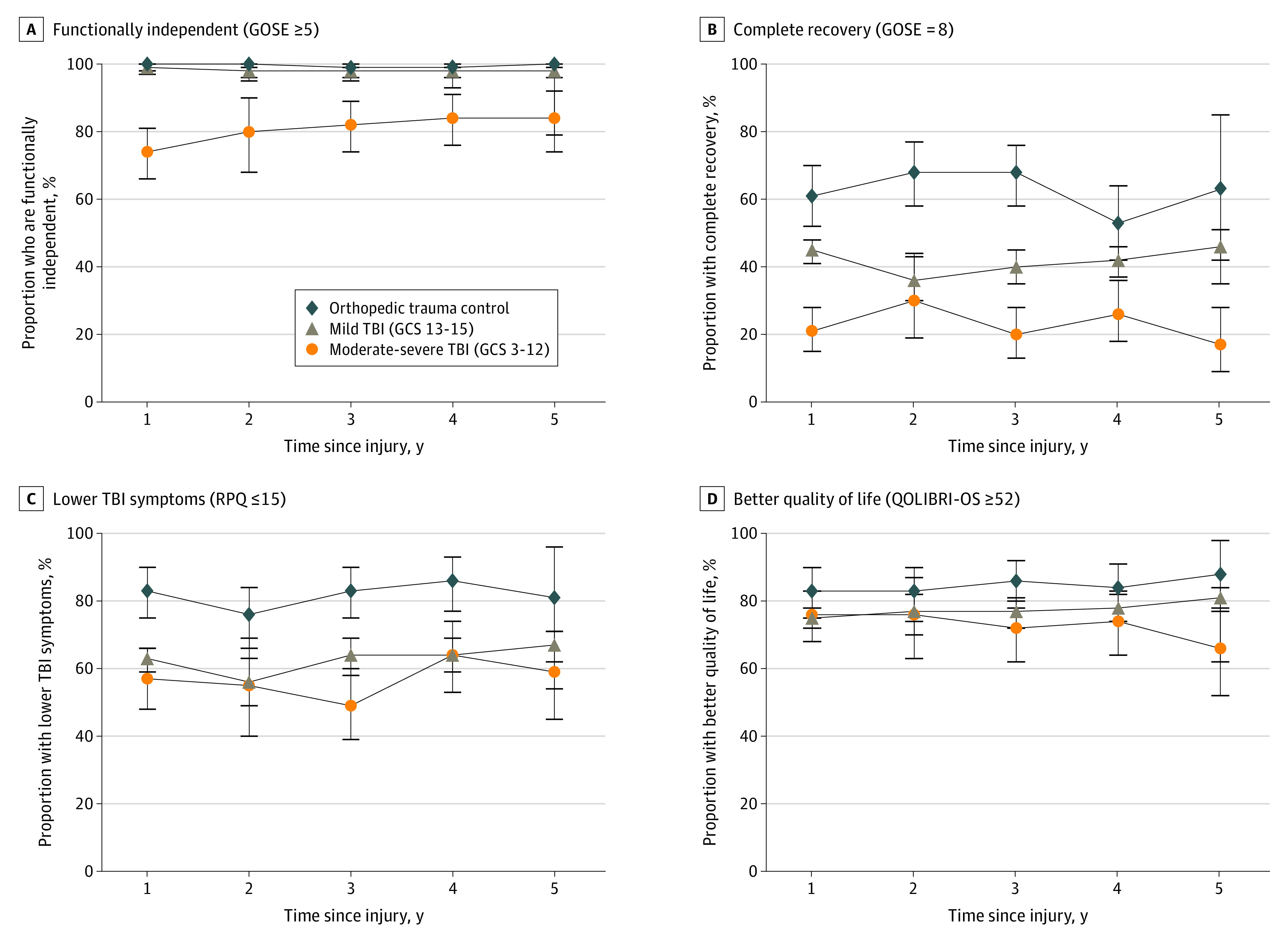
Unweighted Mean Percentages of Patients With Better Outcomes From 1 to 5 Years Postinjury by Group GCS indicates Glasgow Coma Scale; GOSE, Glasgow Outcome Scale-Extended; QOLIBRI-OS, Quality of Life After Brain Injury Scale-Overall Scale; RPQ, Rivermead Post Concussion Symptoms Questionnaire; TBI, traumatic brain injury.

**Table 2.** Multivariable Mixed Effects Logistic Model Depicting Effects of Group, Year, and Other Variables on Odds of Favorable Outcome From 1 to 5 Years Postinjury With Propensity Weighting^a^

Variable	GOSE score ≥5		GOSE score of 8		RPQ score ≤15		QOLIBRI-OS score ≥52	
	OR (95% CI)	*P* value	OR (95% CI)	*P* value	OR (95% CI)	*P* value	OR (95% CI)	*P* value
Group	NA	<.001	NA	<.001	NA	<.001	NA	.004
msTBI vs mTBI	0.01 (0-0.04)	<.001	0.34 (0.24-0.48)	<.001	0.78 (0.54-1.12)	.18	0.81 (0.56-1.17)	.26
msTBI vs OTC	NA	NA	0.13 (0.08-0.21)	<.001	0.22 (0.13-0.37)	<.001	0.43 (0.26-0.72)	.001
mTBI vs OTC	NA	NA	0.39 (0.28-0.56)	<.001	0.28 (0.19-0.43)	<.001	0.54 (0.36-0.81)	.003
Year (per 1 y)	0.81 (0.64-1.03)	.08	1.01 (0.95-1.07)	.72	1.06 (1.00-1.13)	.06	1.05 (0.99-1.13)	.11
Group × year	NA	.005						
msTBI (per 1 y)	1.28 (1.03-1.58)	.02	NA	NA	NA	NA	NA	NA
mTBI (per 1 y)	0.81 (0.64-1.03)	.08	NA	NA	NA	NA	NA	NA
OTC (per 1 y)								
Age (per 10 y)	0.67 (0.55-0.82)	<.001	0.89 (0.82-0.96)	.002	0.98 (0.90-1.06)	.56	0.84 (0.78-0.91)	<.001
Female	0.56 (0.30-1.06)	.07	0.64 (0.50-0.82)	<.001	0.54 (0.42-0.71)	<.001	0.64 (0.49-0.84)	.001
Race	NA	.20	NA	.96	NA	.07	NA	.09
Black (vs White)	0.49 (0.22-1.10)	.08	1.00 (0.70-1.43)	.99	0.72 (0.50-1.03)	.08	0.75 (0.52-1.07)	.11
Other/unknown (vs White)	1.20 (0.31-4.68)	.80	1.07 (0.66-1.73)	.78	1.41 (0.83-2.40)	.21	1.47 (0.83-2.62)	.19
Hispanic ethnicity	0.79 (0.35-1.80)	.58	1.01 (0.73-1.40)	.95	0.83 (0.59-1.17)	.30	1.02 (0.72-1.45)	.91
Non-Medicaid insurance (vs Medicaid or uninsured)	2.35 (1.24-4.46)	.009	1.38 (1.05-1.82)	.02	1.58 (1.19-2.10)	.002	1.84 (1.39-2.45)	<.001
Years of education (per 4 y)	1.11 (0.99-1.25)	.06	1.09 (1.04-1.15)	<.001	1.11 (1.06-1.17)	<.001	1.14 (1.08-1.20)	<.001
Previous TBI	NA	.56	NA	.04	NA	.001	NA	<.001
Yes (vs no)	0.67 (0.31-1.47)	.32	0.68 (0.50-0.92)	.01	0.53 (0.39-0.73)	<.001	0.50 (0.37-0.69)	<.001
Unknown (vs no)	0.73 (0.23-2.33)	.59	1.04 (0.64-1.69)	.88	0.98 (0.58-1.64)	.93	0.99 (0.58-1.71)	.98
Injury cause	NA	.50	NA	.47	NA	.46	NA	.48
Fall (vs MVC)	0.73 (0.34-1.56)	.42	1.20 (0.90-1.59)	.22	1.15 (0.85-1.56)	.38	1.02 (0.75-1.40)	.89
Other/unknown (vs MVC)	0.66 (0.31-1.40)	.28	1.09 (0.80-1.49)	.59	0.91 (0.66-1.27)	.59	0.83 (0.60-1.16)	.28

Abbreviations: GOSE, Glasgow Outcome Scale-Extended; msTBI, moderate-severe traumatic brain injury; mTBI, mild traumatic brain injury; MVC, motor vehicle/traffic crash; NA, not applicable; OTC, orthopedic trauma control; QOLIBRI-OS, Quality of Life After Brain Injury Scale-Overall Scale; RPQ, Rivermead Post Concussion Symptoms Questionnaire; TBI, traumatic brain injury.

^a^ Nonsignificant interactions were dropped from models reported (group × year *P* value was as follows: GOSE, *P* = .72; RPQ, *P* = .91; QOLIBRI-OS, *P* = .20).

The percentage of mTBI and OTC participants with recovery of functional independence (GOSE score 5 or higher) was 98% (95% CI, 96%-99%) to 100% (95% CI, 99%-100%) across time (Figure; eTables 3, 4, and 5 in Supplement 1). Because secondary analyses stratifying mTBI with vs without positive findings on CT scan demonstrated no significant differences between groups, all reported results collapse these groups (eTables 4, 5, and 7 in Supplement 1). Rates were lower for those with msTBI, although most individuals with msTBI achieved independence at 1 year (72%; 95% CI, 64%-79%), and this group showed increasing odds of independence over time (5 years: 80%; 95% CI, 69%-89%; group × year *P* = .005; msTBI per year: OR, 1.28; 95% CI, 1.03-1.58; *P* = .02; mTBI per year: OR, 0.81; 95% CI, 0.64-1.03; *P* = .08) (Table 2). Beyond group and year, odds of functional independence were lower in persons who were older (OR per age 10 years, 0.67; 95% CI, 0.55-0.82) and were higher in those with non-Medicaid insurance (vs Medicaid or no insurance: OR, 2.38; 95% CI, 1.26-4.50). When comparing participants in each group who were dependent vs independent within each GOSE domain (eg, home independence, travel, work, social functioning) across the 5 years of follow-up, domains of independence in the home and independence outside the home (eg, shopping, travel) displayed the largest increases in the percentage of persons demonstrating recovery from 1 to 5 years, as well as the highest rates of complete recovery compared with work, leisure, and relationship functioning, which displayed higher rates of limitations up to 5 years postinjury (Table 3; eTable 8 in Supplement 1).

**Table 3.** Frequencies of Each Glasgow Outcome Scale-Extended Domain

Characteristic	Participants, No./total No. (%) [95% CI]									
	msTBI					mTBI				
	1 y (n = 145)	2 y (n = 55)	3 y (n = 112)	4 y (n = 100)	5 y (n = 69)	1 y (n = 707)	2 y (n = 210)	3 y (n = 350)	4 y (n = 410)	5 y (n = 461)
Independence in the home										
No assistance	111/145 (76) [69-83]	48/55 (87) [75-95]	95/112 (84) [76-91]	86/100 (86) [77-92]	57/69 (82) [71-90]	704/707 (99) [99-100]	206/210 (98) [95-100]	347/350 (99) [97-100]	403/410 (98) [96-99]	454/461 (98) [97-99]
Infrequent assistance	7/145 (5) [2-10]	2/55 (4) [0-12]	4/112 (3) [1-8]	3/100 (3) [0-8]	2/69 (3) [0-10]	2/707 (0) [0-1]	0/210 (0) [0-2]	1/350 (0) [0-2]	3/410 (1) [0-2]	2/461 (0) [0-2]
Frequent assistance	27/145 (19) [13-26]	5/55 (9) [3-20]	14/112 (12) [7-20]	11/100 (11) [6-19]	10/69 (15) [7-25]	1/707 (0) [0-1]	3/210 (2) [0-5]	2/350 (1) [0-2]	4/410 (1) [0-3]	5/461 (1) [0-3]
**Independence in shopping**										
No assistance	108/145 (74) [66-81]	46/55 (82) [70-91]	93/112 (82) [74-89]	85/100 (85) [77-92]	58/69 (83) [72-91]	704/707 (100) [99-100]	204/210 (98) [94-99]	347/350 (99) [97-100]	403/410 (98) [96-99]	453/461 (98) [97-99]
Assistance	37/145 (26) [19-34]	10/55 (18) [9-30]	20/112 (18) [11-26]	15/100 (15) [8-23]	12/69 (17) [9-28]	4/707 (0) [0-1]	5/210 (2) [1-6]	3/350 (1) [0-3]	7/410 (2) [1-4]	8/461 (2) [1-3]
**Independent in traveling**										
No assistance	107/145 (74) [66-81]	45/55 (81) [68-90]	95/112 (84) [76-91]	87/100 (87) [78-93]	58/69 (84) [73-92]	701/707 (99) [98-100]	204/210 (98) [94-99]	345/350 (98) [96-99]	401/410 (98) [96-99]	451/461 (98) [96-99]
Assistance	38/145 (26) [19-34]	11/55 (19) [10-32]	17/112 (16) [9-24]	13/100 (13) [7-22]	11/69 (16) [8-27]	6/707 (1) [0-2]	5/210 (2) [1-6]	5/350 (2) [1-4]	9/410 (2) [1-4]	10/461 (2) [1-4]
**Work**										
No deficit	66/136 (49) [40-57]	29/52 (56) [42-70]	57/102 (55) [45-65]	48/93 (52) [41-62]	31/66 (48) [35-61]	479/595 (80) [77-84]	134/167 (80) [73-86]	239/290 (82) [78-87]	286/339 (84) [80-88]	350/399 (88) [84-91]
Reduced capacity	17/136 (13) [8-20]	8/52 (16) [7-29]	16/102 (16) [9-25]	13/93 (14) [7-22]	9/66 (14) [7-25]	75/595 (13) [10-15]	20/167 (12) [8-18]	29/290 (10) [7-14]	36/339 (10) [7-14]	26/399 (6) [4-9]
Noncompetitive/unable to work	53/136 (39) [30-47]	14/52 (27) [16-42]	29/102 (29) [20-38]	32/93 (35) [25-45]	25/66 (38) [26-51]	42/595 (7) [5-9]	13/167 (8) [4-13]	22/290 (8) [5-11]	17/339 (5) [3-8]	23/399 (6) [4-9]
**Social/leisure functioning**										
No deficit	80/145 (55) [46-63]	33/55 (59) [45-72]	58/112 (51) [42-61]	52/100 (52) [42-62]	32/69 (46) [34-59]	573/707 (81) [78-84]	143/210 (68) [62-75]	252/350 (72) [67-77]	284/410 (69) [65-74]	347/461 (75) [71-79]
A bit less	26/145 (18) [12-25]	9/55 (16) [7-28]	17/112 (15) [9-23]	13/100 (13) [7-21]	7/69 (10) [4-20]	75/707 (11) [8-13]	30/210 (14) [10-20]	50/350 (14) [11-19]	52/410 (13) [10-16]	46/461 (10) [7-13]
Much less	20/145 (14) [8-20]	7/55 (13) [5-25]	20/112 (17) [11-26]	23/100 (23) [15-32]	20/69 (30) [19-42]	42/707 (6) [4-8]	30/210 (14) [10-20]	39/350 (11) [8-15]	51/410 (12) [9-16]	49/461 (11) [8-14]
Unable	20/145 (14) [9-20]	7/55 (13) [5-25]	18/112 (16) [10-24]	12/100 (12) [7-21]	10/69 (14) [7-25]	17/707 (2) [1-4]	7/210 (3) [1-7]	9/350 (2) [1-5]	23/410 (6) [4-8]	19/461 (4) [3-7]
**Family disruption**										
No disruption	86/145 (59) [51-67]	32/55 (58) [44-71]	66/112 (59) [49-68]	60/100 (60) [50-70]	40/69 (57) [45-69]	510/707 (72) [69-75]	128/210 (61) [54-68]	236/350 (67) [62-72]	293/410 (71) [67-76]	345/461 (75) [71-79]
Occasional	12/145 (8) [4-14]	12/55 (22) [12-36]	13/112 (12) [6-19]	18/100 (18) [11-27]	10/69 (15) [7-25]	73/707 (10) [8-13]	39/210 (18) [13-24]	48/350 (14) [10-18]	38/410 (9) [7-12]	59/461 (13) [10-16]
Frequent	34/145 (23) [17-31]	6/55 (10) [4-21]	23/112 (20) [13-29]	14/100 (14) [8-23]	13/69 (18) [10-30]	94/707 (13) [11-16]	27/210 (13) [9-18]	49/350 (14) [11-18]	55/410 (13) [10-17]	47/461 (10) [8-13]
Constant	14/145 (9) [5-15]	5/55 (9) [3-20]	10/112 (9) [4-16]	8/100 (8) [3-15]	7/69 (10) [4-19]	30/707 (4) [3-6]	17/210 (8) [5-12]	17/350 (5) [3-8]	25/410 (6) [4-9]	10/461 (2) [1-4]
**Other disabling symptoms**										
No impact	46/145 (32) [24-40]	18/55 (32) [20-46]	33/112 (29) [21-39]	30/100 (30) [21-40]	21/69 (30) [20-42]	345/706 (49) [45-53]	93/210 (45) [38-52]	152/350 (43) [38-49]	182/410 (44) [40-49]	243/461 (53) [48-57]
Some impact	99/145 (68) [60-76]	38/55 (68) [54-80]	79/112 (71) [61-79]	70/100 (70) [60-79]	48/69 (70) [58-80]	360/706 (51) [47-55]	116/210 (55) [48-62]	198/350 (57) [51-62]	228/410 (56) [51-60]	218/461 (47) [43-52]

Abbreviations: msTBI, moderate-severe traumatic brain injury; mTBI, mild traumatic brain injury; OTC, orthopedic trauma control.

Other outcomes had nonsignificant results of year and group × year (consequently, interaction terms were dropped from the remaining models). Odds of complete functional recovery (GOSE score of 8) were lower for msTBI vs mTBI (17% vs 47% in year 5; group main effect: OR, 0.34; 95% CI, 0.24-0.48) and lower for mTBI vs OTC (47% vs 62% in year 5; group main effect: OR, 0.39 95% CI, 0.28-0.56). Odds of complete functional recovery were also lower vs OTC for persons with older age (per age 10 years: OR, 0.89; 95% CI, 0.82-0.96; *P* = .002), female sex (OR, 0.64; 95% CI, 0.50-0.82; *P* < .001), Medicaid or no insurance (OR for non-Medicaid insurance, 1.38; 95% CI, 1.05-1.82; *P* = .02), lower education (per 4 years: OR, 1.09; 95% CI, 1.04-1.15; *P* < .001), and prior TBI history (yes vs no: OR, 0.68; 95% CI, 0.50-0.92; *P* = .01).

Odds of better symptom outcome (RPQ score 15 or lower) were lower in both mTBI (vs OTC: OR, 0.28; 95% CI, 0.19-0.43) and msTBI (vs OTC: OR, 0.22; 95% CI, 0.13-0.37) groups, and nonsignificant between subgroups (msTBI vs mTBI: OR, 0.78; 95% CI, 0.54-1.12). Odds of better symptom outcome were lower in persons with female sex (OR, 0.54; 95% CI, 0.42-0.71; *P* < .001), Medicaid or no insurance (OR for non-Medicaid insurance, 1.58; 95% CI, 1.19-2.10; *P* = .002), less education (per 4 years: OR, 1.11; 95% CI, 1.06-1.17; *P* < .001), and prior TBI (yes vs no: OR, 0.53; 95% CI, 0.39-0.73; *P* < .001).

Odds of better quality of life (QOLIBRI-OS score 52 or above) were lower in both TBI subgroups relative to OTCs (mTBI: OR, 0.54; 95% CI, 0.36-0.81; msTBI: OR, 0.43; 95% CI, 0.26-0.72) but were comparable with each other (msTBI vs mTBI: OR, 0.81; 95% CI, 0.56-1.17). Odds of better quality of life were lower in persons with older age (per age 10 years: OR, 0.84; 95% CI, 0.78-0.91; *P* < .001), female sex (OR, 0.64; 95% CI, 0.49-0.84; *P* = .001), Medicaid or no insurance (OR for non-Medicaid insurance, 1.84; 95% CI, 1.39-2.45; *P* < .001), lower education (per 4 years: OR, 1.14; 95% CI, 1.08-1.20; *P* < .001), and prior TBI (yes vs no: OR, 0.50; 95% CI, 0.37-0.69; *P* < .001). Persons with msTBI displayed higher rates of death (7 individuals [5.5%] at 5 years) than the mTBI and OTC groups (12 [1.5%] and 1 [0.7%] individuals at 5 years, respectively; log rank *P* = .02) (Table 4).

**Table 4.** Cumulative Mortality From 1 to 5 Years Postinjury Among Persons Believed to Be Living at 1 Year^a^

Year	Deaths, No. (%)			*P* value
	msTBI	mTBI	OTC	
1	0	0	0	.02
2	4 (2.2)	2 (0.2)	1 (0.7)	
3	5 (2.8)	6 (0.7)	1 (0.7)	
4	5 (2.8)	11 (1.3)	1 (0.7)	
5	7 (5.5)	12 (1.5)	1 (0.7)	

Abbreviations: OTC, orthopedic trauma control; msTBI, moderate-severe TBI; mTBI, mild traumatic brain injury.

^a^ Cells report cumulative mortality and number of deaths (cumulative). *P* value from log rank test.

## Discussion

In this prospective, longitudinal study of former level I trauma center patients at 1 to 5 years post-TBI, we found both potential for continued functional recovery and persistently elevated rates of clinical impairment up to 5 years across all TBI severities. Survivors of msTBI demonstrated, on average, increasing odds of recovery of functional independence over time. As defined in this study, independence (ie, GOSE scores of 5 or above) reflected sufficient independence in home activities to be safely left alone for at least 24 hours, as well as the ability to conduct simple shopping tasks and arrange or provide one’s own transportation. This important milestone indicates that persons with TBI, as well as individuals providing support to them, reclaim some autonomy in daily life.

Yet persons who reach this level of function may experience myriad symptoms, cognitive impairments, and physical limitations that disrupt life function and perceived life quality. For example, 83% of msTBI and 53% of mTBI participants reported incomplete functional recovery (ie, GOSE score below 8) at 5 years postinjury, reflecting stable odds of incomplete recovery from 1 to 5 years (ie, no interaction with year) that remained higher than the OTC group (38% at 5 years).^5,15^ Similarly, the proportion of persons with lesser TBI-related symptom burden and better health-related quality of life was stable for all groups over time. The TBI groups showed comparable symptom and quality of life outcomes. Yet, both TBI groups experienced less favorable symptom and quality of life outcomes than the OTC group. These findings illustrate the differing relevance of injury variables across clinical outcomes and highlight the need to investigate the causal factors that drive long-term impairments captured by diverse outcomes.

Our findings align with those of the TBI Model Systems (TBI-MS) study, finding higher rates of functional improvement than decline from 1 to 5 years post-msTBI.^35^ However, the TBI-MS study also illustrated the variable recovery trajectories across individuals, with most demonstrating static functional outcome and a minority declining. Like the TBI-MS study, we also found that despite potential for clinical improvements from 1 to 5 years, persons with msTBI have increased mortality rates relative to mTBI or OTC participants.^36,37^ It is unclear what factors contributed to the outcomes of the msTBI group in our sample. Other studies point to depression, physical comorbidities, cognitive functioning, and age as prognostic of long-term functional and quality of life outcomes after TBI.^8,11,35,38,39^ In light of the relative dearth of rehabilitation services and community supports beyond the first several months postinjury,^12^ our findings justify intensifying efforts to provide longer-term rehabilitation, mental health treatment, and community services for persons with TBI. The elevated rates of incomplete recovery among mTBI participants demonstrates the need for more comprehensive systems of care for level I trauma center patients irrespective of TBI severity and time since injury.

This study also revealed several social determinants of long-term TBI outcomes. Independent of injury group and time since injury, odds of less favorable outcomes manifested in persons who were older, female, and who had less baseline education, Medicaid or no insurance at the time of injury, and histories of prior TBI. Although cause of injury was not associated with outcomes, other research points to assaultive injury as prognostic of some outcomes.^40^ (Our sample had insufficient cases of assault to treat this category separately.) Taken together, our findings support the consensus that TBI recovery is influenced by multiple factors.^14,41,42,43,44,45,46,47,48,49^ Nuanced interrogation of the biological mechanisms by which age and sex affect TBI recovery will fuel the design of more effective treatments. Additionally, efforts to provide follow-up care for TBI must account for difficulties accessing health care that result from insufficient health insurance coverage for many Americans with TBI.^50^

Within individuals with TBI and GCS scores between 13 and 15, abnormal results on a head CT at hospital admission was not associated with clinical outcomes. This is consistent with research finding small or conflicting associations between TBI severity and self-report outcomes of symptoms and quality of life,^17,18,22^ while contributing to knowledge on long-term functional outcome. These findings are likely to reflect the relatively severe grade of CT- GCS 13 to 15 TBI in level I trauma centers (eg, 27% of this subgroup of the TRACK-TBI sample have acute intracranial findings on magnetic resonance imaging around 2 weeks postinjury).^51,52^

### Limitations

This study had several limitations. Our group-level comparisons could not illustrate the varied trajectories of long-term recovery that are known to occur after TBI. Because TRACK-TBI only enrolled level I trauma center patients with TBI who underwent a clinical head CT scan, the findings cannot be generalized beyond these characteristics. Additionally, the study design precluded annual follow-up assessment for every patient during the entire 1-to-5-year follow-up period (eTable 1 in Supplement 1). However, our comparison of weighted and unweighted analysis and sensitivity analyses in a subset of persons followed from 1 to 5 years supports the robustness of our findings. Additionally, because outcomes were gathered from research contact rather than public death databases, mortality may be underestimated.

## Conclusions

This study was unique in its long-term follow-up of persons with mTBI, msTBI, and orthopedic injury. That msTBI survivors at 1 year postinjury experienced both greater increases in functional independence and elevated rates of mortality relative to mTBI and OTC groups demonstrates the need to advance understanding of factors that contribute to long-term TBI outcomes. That mTBI was also associated with persistently elevated rates of adverse clinical outcomes from 1 to 5 years postinjury demonstrates that all level I trauma center patients with TBI should be more closely monitored and treated for injury sequelae, from the acute through chronic recovery period. Finally, the urgency of providing better clinical and community support is paramount.

## Notes

Supplement 1. **eMethods.** Inverse Probability Weighting Methods **eTable 1.** Summary of Participants From the Initial TRACK-TBI Study Considered for the Present Study Who Were Eligible for TRACK-LONG Follow-up Appointments by Year Post-injury and Number Followed **eTable 2.** Overview of Sample of Interest and Predictors of Being Followed 1-5 Years **eTable 3.** Percentage of Individuals With Favorable Recovery From 1-5 Years Post-injury (Unweighted) **eTable 4.** Percentage of Individuals With Favorable Outcome From 1-5 Years Post-injury (Weighted) **eTable 5.** Percentage of Individuals Who Completed Both a Year 1 and Year 4 or 5 Outcome Assessment With Favorable Outcome at Year 1 and Year 4/5 Post-injury (Weighted) **eTable 6.** Mixed Effects Logistic Model Depicting Group, Year, and Group × Year Effects on Favorable Outcome (Without Propensity Weighting or Additional Covariates) **eTable 7.** Multivariable Mixed Effects Logistic Model Depicting Effects of Group, Year, and Other Variables on Odds of Favorable Outcome From 1-5 Years Post-injury (With Propensity Weighting) **eTable 8.** Frequencies of Each Glasgow Outcome Scale-Extended Domain in the Control Group

Supplement 2.
Data Sharing Statement
