# Supplementary material for: Functional Recovery, Symptoms, and Quality of Life 1 to 5 Years After Traumatic Brain Injury
Source: JAMA Netw Open. 2023 Mar 20;6(3):e233660. doi: 10.1001/jamanetworkopen.2023.3660 (PMC10028488; doi:10.1001/jamanetworkopen.2023.3660)
Supplement: Supplement 1. — eMethods. Inverse Probability Weighting Methods eTable 1. Summary of Participants From the Initial TRACK-TBI Study Considered for the Present Study Who Were Eligible for TRACK-LONG Follow-up Appointments by Year Post-injury and Number Followed eTable 2. Overview of Sample of Interest and Predictors of Being Followed 1-5 Years eTable 3. Percentage of Individuals With Favorable Recovery From 1-5 Years Post-injury (Unweighted) eTable 4. Percentage of Individuals With Favorable Outcome From 1-5 Years Post-injury (Weighted) eTable 5. Percentage of Individuals Who Completed Both a Year 1 and Year 4 or 5 Outcome Assessment With Favorable Outcome at Year 1 and Year 4/5 Post-injury (Weighted) eTable 6. Mixed Effects Logistic Model Depicting Group, Year, and Group × Year Effects on Favorable Outcome (Without Propensity Weighting or Additional Covariates) eTable 7. Multivariable Mixed Effects Logistic Model Depicting Effects of Group, Year, and Other Variables on Odds of Favorable Outcome From 1-5 Years Post-injury (With Propensity Weighting) eTable 8. Frequencies of Each Glasgow Outcome Scale-Extended Domain in the Control Group [file jamanetwopen-e233660-s001.pdf]

Supplemental Online Content

Nelson LD, Temkin NR, Barber J, et al; TRACK-TBI Investigators. Functional recovery, symptoms, and quality of life 1 to 5 years after traumatic brain injury. *JAMA Netw Open.* 2023;6(3):e233660. doi:10.1001/jamanetworkopen.2023.3660

eMethods. Inverse Probability Weighting Methods

eTable 1. Summary of Participants From the Initial TRACK-TBI Study Considered for the Present Study Who Were Eligible for TRACK-LONG Follow-up Appointments by Year Post-injury and Number Followed

eTable 2. Overview of Sample of Interest and Predictors of Being Followed 1-5 Years

eTable 3. Percentage of Individuals With Favorable Recovery From 1-5 Years Post-injury (Unweighted)

eTable 4. Percentage of Individuals With Favorable Outcome From 1-5 Years Post-injury (Weighted)

eTable 5. Percentage of Individuals Who Completed Both a Year 1 and Year 4 or 5 Outcome Assessment With Favorable Outcome at Year 1 and Year 4/5 Post-injury (Weighted)

eTable 6. Mixed Effects Logistic Model Depicting Group, Year, and Group × Year Effects on Favorable Outcome (Without Propensity Weighting or Additional Covariates)

eTable 7. Multivariable Mixed Effects Logistic Model Depicting Effects of Group, Year, and Other Variables on Odds of Favorable Outcome From 1-5 Years Post-injury (With Propensity Weighting)

eTable 8. Frequencies of Each Glasgow Outcome Scale-Extended Domain in the Control Group

This supplemental material has been provided by the authors to give readers additional information about their work.

© 2023 Nelson LD et al. *JAMA Network Open.*

eMethods. A boosted regression algorithm was used to model the propensity for having been assessed on any primary outcome during the 2–5-year post-injury interval, utilizing all baseline characteristics listed in Table 1. Boosted regression is a machine-learning technique that uses a series of regression trees to incrementally improve the overall model at each step by modelling the prediction error from the prior tree. The propensity estimates corresponding to the analysis cohort were then inverted to form the statistical weights used in the analysis, standardizing as necessary to ensure that the effective sample size within each severity cohort remained the same.

© 2023 Nelson LD et al. *JAMA Network Open.*

eTable 1. Summary of participants from the initial TRACK-TBI study considered for the present study who were eligible for TRACK-LONG follow-up appointments by year post-injury and number followed.

	Year 1	Year 2	Year 3	Year 4	Year 5
**Eligible**	2661	848	1419	1959	1965
Missed	900	488	849	1351	1405
Followed	1761 (66%)	360 (42%)	570 (40%)	608 (31%)	560 (28%)
**Not Eligible**	---	1813	1242	822	825
Assessment window closed prior to LONG (1/4/19)		1805	1229	682	17
Assessment window closed after export (4/25/22)		0	0	2	599
Subject already had 3 prior LONG assessments		0	0	0	59
Subject previously died		8	13	18	21

Note. The TRACK-TBI study enrolled participants within 24 hours of injury from 2/26/2014 – 7/27/2018 with the initial study designed to follow participants until 1 year post-injury. In 2019, the study obtained funding to initiate annual long-term follow-up phone calls (TRACK-LONG). Participants were eligible to participate if they were living, were at least 2 years post-injury, and had completed at least one GOSE assessment in the first year of follow-up. TRACK-LONG attempted up to 3 annual phone calls per participant.

© 2023 Nelson LD et al. *JAMA Network Open.*

eTable 2. Overview of sample of interest and predictors of being followed 1–5 years[1]

	Full Sample (N=2661)		In Analysis Sample			
			No (N=1465)	Yes (N=1196)	*P* unweighted	*P* weighted
Demographics						
Age, y M (SD)	41.0 (17.0)		41.2 (17.1)	40.8 (16.9)	.67	.70
Sex					.004	.28
Female	847 (32%)		432 (29%)	415 (35%)		
Male	1814 (68%)		1033 (71%)	781 (65%)		
Race[2]						
Black	436 (16%)		278 (19%)	158 (13%)	<.001	.59
White	2034 (76%)		1069 (73%)	965 (81%)		
Other/unknown[3]	191 (7%)		118 (8%)	73 (6%)		
Hispanic ethnicity	565 (21%)		340 (24%)	225 (19%)	.004	.45
Insurance						
Medicaid/uninsured	836 (33%)		505 (38%)	331 (28%)	<.001	.42
Other insured[4]	1671 (67%)		827 (62%)	844 (72%)		
Education, y M (SD)	13.4 (2.9)		13.0 (2.9)	13.9 (2.8)	<.001	<.001
Previous TBI						
No	1926 (80%)		1021 (79%)	905 (81%)	.10	.46
Yes, with hospitalization	298 (12%)		160 (12%)	138 (12%)		
Yes, without hospitalization	188 (8%)		115 (9%)	73 (7%)		
Neurodevelopmental disorder	205 (8%)		109 (7%)	96 (8%)	.61	1.000
Mental health history	592 (22%)		315 (22%)	277 (23%)	.33	.85
Injury characteristics						
Injury Cause						
MVC (occupant)	804 (30%)		474 (32%)	330 (28%)	<.001	.90
MCC	247 (9%)		152 (10%)	95 (8%)		
MVC (cyclist or pedestrian)	376 (14%)		189 (13%)	187 (16%)		
Fall	716 (27%)		383 (26%)	333 (28%)		
Assault	160 (6%)		100 (7%)	60 (5%)		
Other/unknown[5]	358 (13%)		167 (11%)	191 (16%)		
Admission GCS, M (SD)	13.5 (3.3)		13.6 (3.2)	13.4 (3.5)	.41	.49
CT positive (vs. negative)	1022 (44%)		538 (41%)	484 (47%)	.009	.38
Loss of consciousness[6]	1974 (78%)		1090 (78%)	884 (77%)	.39	.20
Post-traumatic amnesia[6]	1722 (72%)		963 (73%)	759 (70%)	.19	.10
AIS head/neck 3+	919 (35%)		491 (34%)	428 (36%)	.24	1.00
Max non-head/neck AIS 3+	530 (20%)		310 (21%)	220 (18%)	.08	.44
Highest level of care						
Emergency department	616 (23%)		340 (23%)	276 (23%)	.67	.87
Inpatient unit	1029 (39%)		556 (38%)	473 (40%)		
Intensive care unit	1016 (38%)		569 (39%)	447 (37%)		
Injury-related litigation	361 (21%)		152 (21%)	209 (21%)	.86	.48

Note. Statistical significance by Mann-Whitney and Fisher's exact tests. p-values reported both unweighted and after propensity-weighting for follow-up. Abbreviations: AIS, Abbreviated

© 2023 Nelson LD et al. *JAMA Network Open.*

Injury Severity score; CT, computed tomography; GCS, Glasgow Coma Scale score; MCC, motorcycle crash; mTBI, mild traumatic brain injury; msTBI, moderate-severe TBI; MVC, motor vehicle crash; OTC, orthopedic trauma control
[1]Data are expressed as No. (%) unless otherwise noted
[2]The source of race and ethnicity (e.g., medical records vs. participant report) was not collected.
[3]Other/unknown race categories and counts (full sample): Alaska Native/Inuit (2), Asian (92), Indian (11), Mixed Race (44), Native Hawaiian/Pacific Islander (7), Unknown (35)
[4]Other insurance categories and counts (full sample): Insurance purchased directly from an insurance company or on the health insurance exchange (this person or family member; 174); Insurance through a current or former employer (of this person or another family member; 1178); Medicare, for people 65 and older, or people with certain disabilities (196); TRICARE, VA or other military health care (47); Other (76)
[5]Injury Cause categories and counts (full sample): Act of mass violence (1), Other (122), Other non-intentional injury (171), Other road traffic accident (42), Suicide attempt (2), Unknown (20)
[6]Witnessed and suspected categories collapsed

© 2023 Nelson LD et al. *JAMA Network Open.*

eTable 3. Percentage of individuals with favorable outcome from 1–5 years post-injury (*unweighted*), displayed as numerator/denominator, % (95% CI)

Outcome	Group	Year 1	Year 2	Year 3	Year 4	Year 5
GOSE ≥ 5	msTBI	109/148<br>74% (66, 81)	45/56<br>80% (68, 90)	91/111<br>82% (74, 89)	87/103<br>84% (76, 91)	59/70<br>84% (74, 92)
	mTBI	714/721<br>99% (98, 100)	199/203<br>98% (95, 99)	335/342<br>98% (96, 99)	414/423<br>98% (96, 99)	464/474<br>98% (96, 99)
	OTC	120/120<br>100% (97, 100)	97/97<br>100% (96, 100)	113/114<br>99% (95, 100)	80/81<br>99% (93, 100)	16/16<br>100% (79, 100)
GOSE = 8	msTBI	31/148<br>21% (15, 28)	17/56<br>30% (19, 44)	22/111<br>20% (13, 28)	27/103<br>26% (18, 36)	12/70<br>17% (9, 28)
	mTBI	321/721<br>45% (41, 48)	74/203<br>36% (30, 43)	137/342<br>40% (35, 45)	176/423<br>42% (37, 46)	219/474<br>46% (42, 51)
	OTC	73/120<br>61% (52, 70)	66/97<br>68% (58, 77)	77/114<br>68% (58, 76)	43/81<br>53% (42, 64)	10/16<br>63% (35, 85)
RPQ ≤ 15	msTBI	70/122<br>57% (48, 66)	28/51<br>55% (40, 69)	49/99<br>49% (39, 60)	59/92<br>64% (53, 74)	34/58<br>59% (45, 71)
	mTBI	465/739<br>63% (59, 66)	114/202<br>56% (49, 63)	216/340<br>64% (58, 69)	266/415<br>64% (59, 69)	313/470<br>67% (62, 71)
	OTC	100/120<br>83% (75, 90)	73/96<br>76% (66, 84)	94/113<br>83% (75, 90)	70/81<br>86% (77, 93)	13/16<br>81% (54, 96)
QOLIBRI-OS ≥ 52	msTBI	93/122<br>76% (68, 83)	39/51<br>76% (63, 87)	71/99<br>72% (62, 80)	69/93<br>74% (64, 83)	38/58<br>66% (52, 78)
	mTBI	554/736<br>75% (72, 78)	155/202<br>77% (70, 82)	258/337<br>77% (72, 81)	325/417<br>78% (74, 82)	379/470<br>81% (77, 84)
	OTC	100/120<br>83% (75, 90)	80/96<br>83% (74, 90)	97/113<br>86% (78, 92)	68/81<br>84% (74, 91)	14/16<br>88% (62, 98)

Note. GOSE, Glasgow Outcome Scale-Extended; mTBI, mild traumatic brain injury; msTBI, moderate-severe traumatic brain injury; OTC, orthopedic trauma; QOLIBRI-OS, Quality of Life After Brain Injury Scale-Overall Scale; RPQ, Rivermead Post Concussion Symptoms Questionnaire

© 2023 Nelson LD et al. *JAMA Network Open.*

eTable 4. Percentage of individuals with favorable recovery from 1–5 years post-injury (*weighted*), displayed as numerator/denominator, % (95% CI)

Outcome	Group	Year 1	Year 2	Year 3	Year 4	Year 5
GOSE ≥ 5	msTBI	105/145<br>72% (64, 79)	44/55<br>79% (66, 89)	92/112<br>82% (73, 88)	83/100<br>83% (75, 90)	55/69<br>80% (69, 89)
	mTBI all	700/707<br>99% (98, 100)	204/210<br>98% (94, 99)	343/350<br>98% (96, 99)	401/410<br>98% (96, 99)	451/461<br>98% (96, 99)
	mTBI CT+	244/249<br>98% (95, 99)	70/73<br>96% (89, 99)	123/127<br>97% (92, 99)	146/153<br>96% (91, 98)	141/146<br>97% (92, 99)
	mTBI CT-	439/439<br>100% (99, 100)	120/121<br>99% (95, 100)	208/210<br>99% (96, 100)	248/251<br>99% (96, 100)	305/311<br>98% (96, 99)
	OTC	113/113<br>100% (97, 100)	96/96<br>100% (96, 100)	110/111<br>99% (95, 100)	74/75<br>99% (92, 100)	16/16<br>100% (79, 100)
GOSE = 8	msTBI	28/145<br>19% (13, 26)	16/55<br>29% (18, 43)	22/112<br>19% (12, 28)	25/100<br>25% (17, 34)	12/69<br>17% (9, 29)
	mTBI all	311/707<br>44% (40, 48)	69/210<br>33% (26, 40)	138/350<br>39% (34, 45)	165/410<br>40% (35, 45)	215/461<br>47% (42, 51)
	mTBI CT+	91/249<br>36% (30, 43)	29/73<br>40% (29, 52)	47/127<br>37% (29, 46)	57/153<br>37% (29, 45)	69/146<br>47% (39, 56)
	mTBI CT-	216/439<br>49% (44, 54)	35/121<br>29% (21, 38)	87/210<br>41% (35, 48)	104/251<br>42% (35, 48)	145/311<br>47% (41, 52)
	OTC	68/113<br>60% (50, 69)	65/96<br>67% (57, 76)	75/111<br>68% (58, 76)	39/75<br>52% (40, 64)	10/16<br>62% (34, 85)
RPQ ≤ 15	msTBI	69/120<br>58% (48, 67)	28/50<br>56% (41, 70)	49/99<br>50% (39, 60)	56/88<br>64% (53, 74)	32/55<br>59% (45, 72)
	mTBI all	445/724<br>62% (58, 65)	115/208<br>55% (48, 62)	213/349<br>61% (56, 66)	253/402<br>63% (58, 68)	302/457<br>66% (62, 70)
	mTBI CT+	156/248<br>63% (56, 69)	41/71<br>57% (45, 69)	79/125<br>63% (54, 72)	97/147<br>66% (57, 73)	102/143<br>72% (63, 79)
	mTBI CT-	283/458<br>62% (57, 66)	66/121<br>54% (45, 63)	123/210<br>59% (52, 66)	153/250<br>61% (55, 67)	197/310<br>64% (58, 69)

© 2023 Nelson LD et al. *JAMA Network Open.*

	OTC	94/113<br>83% (75, 89)	69/96<br>72% (62, 81)	91/110<br>83% (74, 89)	65/75<br>86% (77, 93)	13/16<br>84% (57, 97)
QOLIBRI-OS ≥ 52	msTBI	90/120<br>75% (67, 83)	38/50<br>75% (61, 87)	72/99<br>73% (63, 81)	66/89<br>74% (64, 83)	36/55<br>66% (51, 78)
	mTBI all	539/721<br>75% (71, 78)	161/208<br>77% (71, 83)	265/346<br>77% (72, 81)	310/403<br>77% (73, 81)	364/457<br>80% (76, 83)
	mTBI CT+	188/248<br>76% (70, 81)	53/71<br>75% (63, 85)	93/125<br>75% (66, 82)	111/147<br>75% (68, 82)	115/143<br>80% (73, 86)
	mTBI CT-	340/457<br>74% (70, 78)	95/121<br>78% (69, 85)	160/208<br>77% (71, 83)	197/251<br>78% (73, 83)	246/310<br>79% (75, 84)
	OTC	95/113<br>84% (76, 90)	79/96<br>83% (74, 90)	95/110<br>86% (78, 92)	62/75<br>83% (72, 91)	13/16<br>86% (58, 98)

Note. Some denominators differ slightly from unweighted because weighting was done for inclusion in the analysis sample rather than inclusion for the sample at year 1, 2, 3, 4, and 5 separately. GOSE, Glasgow Outcome Scale-Extended; mTBI, mild traumatic brain injury; msTBI, moderate-severe traumatic brain injury; OTC, orthopedic trauma; QOLIBRI-OS, Quality of Life After Brain Injury Scale-Overall Scale; RPQ, Rivermead Post Concussion Symptoms Questionnaire

© 2023 Nelson LD et al. *JAMA Network Open.*

eTable 5. Percentage of individuals *who completed both a Year 1 and Year 4 or 5 outcome assessment (n = 759)* with favorable outcome at Year 1 and Year 4/5 post-injury (*weighted*), displayed as numerator/denominator, % (95% CI)

Outcome	Group	Year 1	Year 4/5 combined (Year 5 if available, otherwise Year 4)
GOSE ≥ 5	msTBI	80/107<br>75% (65, 83)	87/107<br>81% (72, 88)
	mTBI all	576/583<br>99% (97, 99)	573/583<br>98% (97, 99)
	mTBI CT+	194/200<br>97% (94, 99)	194/200<br>97% (94, 99)
	mTBI CT-	378/378<br>100% (99, 100)	372/378<br>99% (97, 99)
	OTC	68/68<br>100% (95, 100)	67/68<br>99% (91, 100)
GOSE = 8	msTBI	20/107<br>18% (11, 27)	23/107<br>21% (14, 30)
	mTBI all	264/583<br>45% (41, 49)	251/583<br>43% (39, 47)
	mTBI CT+	67/200<br>33% (27, 40)	81/200<br>40% (34, 48)
	mTBI CT-	197/378<br>52% (47, 57)	167/378<br>44% (39, 49)
	OTC	40/68<br>59% (47, 71)	32/68<br>47% (35, 60)
RPQ ≤ 15	msTBI	8/86<br>9% (4, 17)	7/89<br>8% (3, 16)
	mTBI all	68/578<br>12% (9, 15)	79/575<br>14% (11, 17)
	mTBI CT+	22/197	30/193

© 2023 Nelson LD et al. *JAMA Network Open.*

		11% (7, 16)	16% (11, 21)
	mTBI CT-	46/377<br>12% (9, 16)	49/377<br>13% (10, 17)
	OTC	15/68<br>23% (13, 34)	14/68<br>20% (12, 32)
QOLIBRI-OS ≥ 52	msTBI	46/86<br>53% (42, 64)	57/89<br>63% (52, 73)
	mTBI all	366/578<br>63% (59, 67)	374/575<br>65% (61, 69)
	mTBI CT+	120/197<br>61% (54, 68)	131/193<br>68% (60, 74)
	mTBI CT-	244/377<br>65% (60, 70)	242/377<br>64% (59, 69)
	OTC	53/68<br>79% (67, 88)	57/68<br>84% (73, 92)

Note. GOSE, Glasgow Outcome Scale-Extended; mTBI, mild traumatic brain injury; msTBI, moderate-severe traumatic brain injury; OTC, orthopedic trauma; QOLIBRI-OS, Quality of Life After Brain Injury Scale-Overall Scale; RPQ, Rivermead Post Concussion Symptoms Questionnaire

© 2023 Nelson LD et al. *JAMA Network Open.*

eTable 6. Mixed effects logistic model depicting Group, Year and Group x Year effects on favorable outcome (*without propensity weighting or additional covariates*)

Group Comparison	GOSE ≥ 5		GOSE = 8		RPQ ≤ 15		QOLIBRI-OS ≥ 52	
	OR (95% CI)	P	OR (95% CI)	P	OR (95% CI)	P	OR (95% CI)	P
Group	---	<.001	---	<.001	---	<.001	---	.007
msTBI vs. mTBI	0.02 (0.01, 0.07)	<.001	0.33 (0.23, 0.46)	<.001	0.75 (0.54, 1.06)	.10	0.82 (0.58, 1.16)	.26
msTBI vs. OTC			0.13 (0.08, 0.20)	<.001	0.24 (0.15, 0.39)	<.001	0.47 (0.29, 0.77)	.002
mTBI vs. OTC			0.38 (0.27, 0.54)	<.001	0.32 (0.21, 0.47)	<.001	0.58 (0.39, 0.85)	.006
Year (per +1yr)	0.83 (0.66, 1.03)	.10	0.99 (0.94, 1.05)	.78	1.04 (0.99, 1.11)	.14	1.05 (0.98, 1.11)	.17
Group x Year	---	.007						
msTBI (per +1yr)	1.24 (1.02, 1.50)	.03						
mTBI (per +1yr)	0.83 (0.66, 1.03)	.10						

Note. Nonsignificant interactions were dropped from models reported (Group x Year P value was as follows: GOSE = 8 P = .75, RPQ ≤ 15 P = .90, QOLIBRI-OS ≥ 52 P = .13). GOSE, Glasgow Outcome Scale-Extended; mTBI, mild traumatic brain injury; msTBI, moderate-severe traumatic brain injury; OTC, orthopedic trauma; QOLIBRI-OS, Quality of Life After Brain Injury Scale-Overall Scale; RPQ, Rivermead Post Concussion Symptoms Questionnaire

© 2023 Nelson LD et al. *JAMA Network Open.*

eTable 7. Multivariable mixed effects logistic model depicting effects of Group *(Four groups: msTBI, CT+ mTBI, CT- mTBI, and OTC)*, Year, and other variables on odds of favorable outcome from 1–5 years post-injury (*with propensity weighting*)

	GOSE ≥ 5[1]		GOSE = 8		RPQ ≤ 15		QOLIBRI-OS ≥ 52	
	OR (95% CI)	P	OR (95% CI)	P	OR (95% CI)	P	OR (95% CI)	P
Group	---	<.001	---	<.001	---	<.001	---	.011
msTBI vs. mTBI CT+	0.07 (0.03, 0.16)	<.001	0.40 (0.27, 0.60)	<.001	0.72 (0.48, 1.10)	.13	0.79 (0.52, 1.21)	.28
msTBI vs. mTBI CT-			0.30 (0.21, 0.44)	<.001	0.80 (0.55, 1.17)	.26	0.80 (0.55, 1.18)	.26
mTBI vs. OTC			0.14 (0.08, 0.22)	<.001	0.22 (0.13, 0.37)	<.001	0.43 (0.26, 0.72)	.001
mTBI CT+ vs. mTBI CT-			0.75 (0.56, 1.01)	.06	1.11 (0.81, 1.51)	.51	1.02 (0.74, 1.40)	.93
mTBI CT+ vs. OTC			0.34 (0.23, 0.50)	<.001	0.31 (0.19, 0.48)	<.001	0.55 (0.35, 0.86)	.009
mTBI CT- vs. OTC			0.45 (0.31, 0.65)	<.001	0.28 (0.18, 0.43)	<.001	0.54 (0.35, 0.83)	.005
Year (per +1yr)	1.14 (0.96, 1.36)	.13	1.00 (0.95, 1.06)	.91	1.06 (0.99, 1.12)	.08	1.05 (0.99, 1.13)	.12
Age (per +10yrs)	0.66 (0.52, 0.84)	.001	0.90 (0.83, 0.97)	.006	0.97 (0.89, 1.05)	.43	0.83 (0.76, 0.90)	<.001
Female	0.60 (0.29, 1.25)	.17	0.63 (0.49, 0.81)	<.001	0.55 (0.42, 0.71)	<.001	0.64 (0.49, 0.84)	.001
Race	---	.46	---	.97	---	.11	---	.13
Black (vs. White)	0.52 (0.18, 1.47)	.22	0.99 (0.69, 1.43)	.97	0.74 (0.51, 1.07)	.11	0.76 (0.53, 1.10)	.14
Other/unknown (vs. White)	1.04 (0.25, 4.32)	.96	1.06 (0.65, 1.73)	.82	1.36 (0.79, 2.32)	.27	1.42 (0.79, 2.53)	.24

© 2023 Nelson LD et al. *JAMA Network Open.*

Hispanic ethnicity	0.74<br>(0.30, 1.84)	.52	1.04<br>(0.74, 1.46)	.82	0.86<br>(0.61, 1.22)	.40	0.99<br>(0.69, 1.42)	.96
Non-Medicaid insurance (vs. Medicaid/uninsured)	1.97<br>(0.95, 4.10)	.07	1.40<br>(1.06, 1.85)	.02	1.58<br>(1.19, 2.11)	.002	1.85<br>(1.39, 2.46)	<.001
Years of education (per +4yrs)	1.09<br>(0.97, 1.23)	.16	1.10<br>(1.05, 1.15)	<.001	1.11<br>(1.06, 1.17)	<.001	1.14<br>(1.08, 1.20)	<.001
Previous TBI	---	.27	---	.03	---	.001	---	<.001
Yes (vs. no)	0.48<br>(0.20, 1.19)	.11	0.66<br>(0.48, 0.90)	.008	0.53<br>(0.39, 0.74)	<.001	0.50<br>(0.37, 0.69)	<.001
Unknown (vs. no)	1.06<br>(0.26, 4.30)	.93	1.03<br>(0.63, 1.70)	.90	1.00<br>(0.59, 1.69)	>.99	1.05<br>(0.60, 1.82)	.87
Injury Cause	---	.91	---	.33	---	.40	---	.49
Fall (vs. MVC)	1.14<br>(0.48, 2.69)	.77	1.24<br>(0.93, 1.67)	.15	1.18<br>(0.86, 1.61)	.31	1.05<br>(0.76, 1.44)	.78
Other/Unknown (vs. MVC)	1.20<br>(0.50, 2.88)	.69	1.14<br>(0.83, 1.57)	.43	0.92<br>(0.65, 1.28)	.60	0.84<br>(0.60, 1.18)	.31

Note. Nonsignificant interactions were dropped from models reported (Group x Year P value was as follows: GOSE = 8 P = .51, RPQ ≤ 15 P = .44, QOLIBRI-OS ≥ 52 P = .98). mTBI, mild traumatic brain injury; msTBI, moderate-severe traumatic brain injury; MVC, motor vehicle/traffic crash; OTC, orthopedic trauma; TBI, traumatic brain injury

[1]The OTC and mTBI CT- groups were dropped from the model due to model estimation issues resulting from no variability in the outcome at one or more timepoints.

© 2023 Nelson LD et al. *JAMA Network Open.*

eTable 8. Frequencies of Each Glasgow Outcome Scale-Extended Domain in the Control Group

Characteristic	Participants, No./total No. (%) [95% CI]				
	OTC				
	1 y (n = 113)	2 y (n = 96)	3 y (n = 111)	4 y (n = 75)	5 y (n = 16)
**Independence in the home**					
No assistance	113/113 (100) [97-100]	96/96 (100) [96-100]	110/111 (99) [95-100]	74/75 (99) [92-100]	16/16 (100) [79-100]
Infrequent assistance	0/113 (0) [0-3]	0/96 (0) [0-4]	1/111 (1) [0-5]	1/75 (1) [0-8]	0/16 (0) [0-21]
Frequent assistance	0/113 (0) [0-3]	0/96 (0) [0-4]	0/111 (0) [0-3]	0/75 (0) [0-5]	0/16 (0) [0-21]
**Independence in shopping**					
No assistance	113/113 (100) [97-100]	96/96 (100) [96-100]	110/111 (99) [95-100]	74/75 (99) [92-100]	16/16 (100) [79-100]
Assistance	0/113 (0) [0-3]	0/96 (0) [0-4]	1/111 (1) [0-5]	1/75 (1) [0-8]	0/16 (0) [0-21]
**Independent in traveling**					
No assistance	113/113 (100) [97-100]	96/96 (100) [96-100]	110/111 (99) [95-100]	74/75 (99) [92-100]	16/16 (100) [79-100]
Assistance	0/113 (0) [0-3]	0/96 (0) [0-4]	1/111 (1) [0-5]	1/75 (1) [0-8]	0/16 (0) [0-21]
**Work**					
No deficit	83/103 (81) [72-88]	75/89 (85) [75-91]	93/102 (91) [84-96]	61/70 (87) [76-94]	13/15 (87) [58-99]
Reduced capacity	16/103 (16) [9-24]	9/89 (10) [4-18]	4/102 (4) [1-10]	6/70 (9) [3-18]	1/15 (8) [0-34]
Noncompetitive/unable to work	4/103 (4) [1-10]	5/89 (5) [2-13]	5/102 (5) [1-11]	3/70 (4) [1-12]	1/15 (6) [0-33]
**Social/leisure functioning**					
No deficit	97/113 (85) [77-91]	74/96 (77) [67-85]	90/111 (81) [72-88]	57/75 (75) [64-85]	12/16 (75) [46-93]
A bit less	12/113 (11) [5-18]	13/96 (14) [7-22]	10/111 (9) [5-16]	14/75 (19) [11-30]	3/16 (20) [4-47]
Much less	3/113 (3) [1-8]	8/96 (9) [4-16]	9/111 (8) [4-15]	3/75 (4) [1-12]	1/16 (5) [0-31]
Unable	2/113 (2) [0-6]	1/96 (1) [0-6]	2/111 (2) [0-6]	1/75 (1) [0-8]	0/16 (0) [0-21]

© 2023 Nelson LD et al. *JAMA Network Open.*

**Family disruption**					
No disruption	107/113 (94) [88-98]	89/96 (92) [85-97]	96/111 (87) [79-93]	64/75 (85) [75-92]	16/16 (100) [79-100]
Occasional	4/113 (3) [1-9]	4/96 (4) [1-10]	7/111 (6) [3-13]	5/75 (7) [2-15]	0/16 (0) [0-21]
Frequent	3/113 (3) [0-8]	4/96 (4) [1-10]	4/111 (3) [1-8]	6/75 (8) [3-17]	0/16 (0) [0-21]
Constant	0/113 (0) [0-3]	0/96 (0) [0-4]	4/111 (4) [1-9]	0/75 (0) [0-5]	0/16 (0) [0-21]
**Other disabling symptoms**					
No impact	88/113 (78) [69-85]	75/96 (78) [69-86]	87/111 (79) [70-86]	56/75 (75) [64-84]	12/16 (75) [46-93]
Some impact	25/113 (22) [15-31]	21/96 (22) [14-31]	24/111 (21) [14-30]	19/75 (25) [16-36]	4/16 (25) [7-54]

© 2023 Nelson LD et al. *JAMA Network Open.*
