# Supplementary material for: Functional Recovery, Symptoms, and Quality of Life 1 to 5 Years After Traumatic Brain Injury
Source: JAMA Netw Open. 2023 Mar 20;6(3):e233660. doi: 10.1001/jamanetworkopen.2023.3660 (PMC10028488; doi:10.1001/jamanetworkopen.2023.3660)
Supplement: Supplement 2. — Data Sharing Statement [file jamanetwopen-e233660-s002.pdf]

Data Sharing Statement

Nelson. Functional Recovery, Symptoms, and Quality of Life 1 to 5 Years After Traumatic Brain Injury. *JAMA Netw Open*. Published March 20, 2023. doi:10.1001/jamanetworkopen.2023.3660

Data
Data available: No

Additional Information
Explanation for why data not available: Data from the parent TRACK-TBI study from enrollment through 12-month follow-up are available through the Federal Interagency Traumatic Brain Injury Research (FITBIR) data repository. TRACK-TBI LONG procedures and data collection forms may be found at https://tracktbi.ucsf.edu/researchers. Investigators interested in the longer-term outcome data can submit a Data Collaboration Request to the TRACK-TBI Executive Committee through the process outlined on its website (https://tracktbi.ucsf.edu/collaboration-opportunities).
